# The C‐terminal region of KIF26B is indispensable for nephron progenitor condensation and kidney formation in mice

**DOI:** 10.1002/2211-5463.70260

**Published:** 2026-05-18

**Authors:** Yuta Yamamura, Kengo Furuichi, Hiromitsu Akamatsu, Naoki Yamamoto, Sako Keisuke, Akihiko Koshino, Taichiro Minami, Megumi Oshima, Akinori Hara, Norihiko Sakai, Miho Shimizu, Kenta Takahashi, Minako Yamamura, Shin‐ichi Horike, Takiko Daikoku, Kenichi Harada, Yohei Shinmyo, Hiroshi Kawasaki, Takashi Wada, Yasunori Iwata

**Affiliations:** ^1^ Department of Nephrology and Rheumatology Kanazawa University Japan; ^2^ Department of Nephrology Kanazawa Medical University Uchinada Japan; ^3^ Department of Human Pathology Kanazawa University Japan; ^4^ Division of Integrated Omics Research, Research Center for Experimental Modeling of Human Disease Kanazawa University Japan; ^5^ Division of Animal Disease Model, Research Center for Experimental Modeling of Human Disease Kanazawa University Japan; ^6^ Department of Neurophysiology Hamamatsu University School of Medicine Japan; ^7^ Department of Medical Neuroscience, Graduate School of Medical Sciences Kanazawa University Japan; ^8^ Sapiens Life Sciences, Evolution and Medicine Research Center Kanazawa University Japan

**Keywords:** CAKUT, kidney development, Kif26b, PAX2, renal coloboma syndrome

## Abstract

Loss of Kif26b function causes renal agenesis or hypoplasia in mice, and a KIF26B mutation resulting in deletion of the C‐terminal region has been identified in a human patient with a congenital kidney disorder. However, the role of this region during kidney development is unclear; thus, to investigate this, we generated mice lacking the C‐terminal region of KIF26B (Kif26b‐C) using CRISPR/Cas9. Homozygous Kif26b‐C mice showed bilateral renal agenesis, resembling Kif26b knockout mice. Histological analysis revealed loss of the nephrogenic zone and impaired nephron progenitor condensation at embryonic day 18.5. *In situ* hybridization showed reduced Gdnf in the metanephric mesenchyme and reduced Wnt11 in ureteric buds, indicating disruption of the Gdnf‐Wnt11 signaling loop. These findings suggest a key role for the KIF26B C‐terminal region in kidney morphogenesis.

## Graphical abstract

KIF26B plays an important role in kidney development. We engineered mice lacking the C‐terminal region of KIF26B and found severe kidney defects, including bilateral renal agenesis, similar to full Kif26b knockout mice. The mutation disrupted nephron progenitor condensation and reduced Gdnf‐Wnt11 signaling, showing that the KIF26B C‐terminal region is essential for normal kidney morphogenesis.

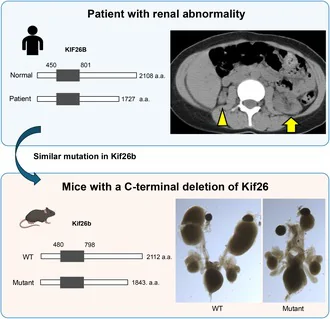

**Abbreviations**
GDNF: glial cell line‐derived neurotrophic factor
gRNA: guide‐RNA
H&E: hematoxylin and eosin
Kif26b: Kinesin Family Member 26B
Myh10: myosin heavy chain 10
RCS: renal coloboma syndrome

Kinesin Family Member 26B (Kif26b) is a member of the kinesin family and plays an essential role in kidney development [1, 2, 3]. Homozygous Kif26b knockout mice exhibit bilateral or unilateral kidney agenesis or hypoplasia [3]. In humans, we previously identified the first pathogenic mutation in KIF26B as the cause of renal coloboma syndrome (RCS), a rare genetic kidney disorder [4]. These findings indicate that KIF26B is crucial for kidney development.

Despite these insights, the precise role of Kif26b in kidney development remains incompletely understood. Kif26b consists of a motor domain and other regions and is primarily expressed in the metanephric mesenchyme, where it contributes to the adhesion of metanephric mesenchymal cells adjacent to the ureteric buds [3]. Interestingly, mice with mesenchyme‐specific deletion of Kif26b develop apparently normal kidneys and survive into adulthood [5], in contrast to global Kif26b knockout mice, which exhibit severe renal defects and perinatal lethality [3]. We previously reported a human KIF26B mutation causing deletion of the C‐terminal region, suggesting the functional importance of this domain [4]. *In vitro* studies have further suggested that the C‐terminal region may interact with myosin heavy chain 10 (Myh10) [3, 6], highlighting the potential significance of this region for kidney development.

In this study, we aimed to investigate the role of the C‐terminal region of Kif26b in kidney development. We generated Kif26b mutant mice lacking the C‐terminal region and found that these mice exhibit kidney abnormalities similar to those observed in RCS patients. Furthermore, these mice showed impaired condensation of nephron progenitor cells and reduced expression of glial cell line‐derived neurotrophic factor (Gdnf) in the metanephric mesenchyme and Wnt11 in the ureteric buds. Our findings provide new insights into the role of the Kif26b C‐terminal domain in kidney development and may provide a framework to understand congenital kidney hypoplasia.

## Materials and methods

### Ethics statement, study approval

All animal experiments were approved by the Institute for Experimental Animals, Kanazawa University Advanced Research Center (registration number, AP21‐009‐02) and conducted in accordance with the institutional guidelines and the ARRIVE 2.0 guidelines. Written informed consent was obtained from the patient for use of clinical data.

### Mouse management

Mice were housed in a specific pathogen‐free facility under controlled conditions with a 12‐h light/dark cycle. Food and water were provided *ad libitum*. All efforts were made to minimize animal suffering. Pregnant mice were monitored daily, and procedures were performed by trained personnel to reduce distress. Pregnant mice were euthanized by CO_2_ inhalation followed by cervical dislocation in accordance with institutional guidelines. Fetal tissues were evaluated for morphology, organ development, histology, and molecular analysis. Each litter was considered as an independent biological replicate.

### Sex as a biological variable

For animal experiments, sex was not included as a biological variable in this study design or analysis. Both male and female mice were used.

### Kif26b knockout mice

C57BL/6J mice were purchased from the Jackson Laboratory Japan, Inc. (Kanagawa, Japan). Kif26b hetero knockout mice were provided by RIKEN with the permission of Dr. Ryuichi Nishinakamura, Kumamoto University, who is the founder of the knockout mouse.

### Kif26b mutant mice (C‐terminal deletion)

Kif26b mutant mice have a defect near the C‐terminal region of Kif26b, which is similar mutation with the case we reported previously [4]. Kif26b mutant mice with a C‐terminal deletion were generated by CRISPR/Cas9‐mediated targeting strategy. Briefly, fertilized eggs were collected from C57BL/6J mice, and Cas9 mRNA, Kif26b guide‐RNA (gRNA), and single‐stranded oligodeoxynucleotide (ssODN) were injected into fertilized eggs. To model the functional consequence of the patient variant (loss of the C‐terminal region) rather than reproduce the exact nucleotide breakpoint, a premature stop codon was introduced approximately 120 bases upstream of the human deletion site to ensure a consistent C‐terminal truncation. The deletion of the C‐terminal region of Kif26b was confirmed by Sanger sequence using standard protocol. The genotypes of breeding mice were analyzed using the following primers and product sizes (F: AACAGGGCCAGCCCTCAACAC‐3′, R: 5′‐AGAAGCTGAGGGTTTTGGTGGT‐3′, Homozygous: two bands with 270 and 210 bp, Heterozygous: three bands with 480, 270, and 210 bp, WT: one band with 480 bp) as shown in Fig. S1A.

### Kif26b C‐terminal GFP‐tagged transgenic mice

Transgenic mice expressing the GFP‐tagged C‐terminal region of Kif26b were generated by a conventional transgenic approach. As described in our previous paper [7], the expression plasmid encoding GFP–Kif26b‐C was constructed by replacing the EF1α promoter in the pLEX307 GFP–Kif26b‐C vector (Addgene, Cambridge, MA, USA) [8] with the CAG promoter to achieve robust expression in mammalian tissues. The promoter replacement was performed using the NEBuilder HiFi DNA Assembly Master Mix (New England Biolabs, Ipswich, MA, USA) following the manufacturer's instructions. The fertilized eggs were collected from C57BL/6N from SLC (Shizuoka, Japan), and the CAG promoter‐GFP‐Kif26b‐C DNA fragment was injected into the pronuclei. The genotypes of breeding mice were performed by targeting the GFP sequence (Product size: 229 bps, GFP‐F: GGTGAACTTCAAGATCCGCC, GFP‐R: CTTGTACAGCTCGTCCATGC).

### Immunohistochemical analyses

For immunostaining, embryonic and adult kidneys were dissected and fixed in 10% neutral buffered formalin (pH 7.2). Tissues were embedded in paraffin and sliced into 5‐μm‐thick sections. Hematoxylin and eosin (H&E) staining and immunostaining for Pax2, Integrin α8, and CK8 were performed with rabbit anti‐Integrinα8 polyclonal antibody (HPA003432; Sigma‐Aldrich, St.Louis, MI, USA), rabbit anti‐Pax2 monoclonal antibody (ab79389; Abcam, Cambridge, UK), and rat anti‐Cytokeratin 8 monoclonal antibody (MABT329; Merck Millipore, Burlington, MA, USA).

### mRNA *in situ* hybridization

mRNA *in situ* hybridization was performed on formalin‐fixed, paraffin‐embedded sections. RNAscope 2.5 assay (ACDBio, Newark, CA, USA) was used according to the manufacturer's instructions. Recommended probes for Gdnf (421951; ACDBio) and Wnt11 (405021; ACDBio) were used.

### Western blot analysis

Samples were solubilized in 1× RIPA lysis buffer (Merck Millipore) supplemented with protease inhibitor (Roche, Basel, Switzerland). Tissue lysates were separated by SDS/polyacrylamide gel electrophoresis (Wako, Osaka, Japan) and transferred to polyvinylidene difluoride membranes (Merck Millipore), followed by immunoblotting. The following antibodies were used: Rabbit anti‐Kif26b polyclonal antibody (17422‐1‐AP; Proteintech, Rosemont, IL, USA) and mouse anti‐Gapdh monoclonal antibody (sc‐32233; Santa Cruz, Santa Cruz, CA, USA).

## Results

### The C‐terminal region of Kif26b is essential for kidney development in mice

We previously identified a novel KIF26B mutation in a patient with RCS who lacked a PAX2 mutation [4]. The patient was a 31‐year‐old female with no family history of genetic kidney disease, presenting with kidney dysfunction, renal hypoplasia, and optic nerve coloboma. Computed tomography revealed severe bilateral renal hypoplasia (Fig. 1A). To investigate the critical region of Kif26b in kidney development, we generated Kif26b mutant mice with a C‐terminal deletion using CRISPR/Cas9 genome editing (Kif26b‐C mice), modeling the human KIF26B mutation. This allele was designed to model the functional consequence of the patient variant (C‐terminal truncation) rather than reproduce the exact nucleotide breakpoint. The Kif26b‐C mice harbored a premature stop codon approximately 120 base pairs upstream of the human mutation site (Fig. 1B,C), and the mutation was confirmed by DNA sequencing (Fig. 1D). We further confirmed that KIF26B protein levels were not markedly reduced, whereas the protein size was slightly decreased in embryonic kidneys from Kif26b‐C mice at E14.5 (Fig. S1B). In addition, the KIF26B band detected by western blotting disappeared in limb buds from Kif26b knockout mice, confirming antibody specificity (Fig. S1C).

**Fig. 1:**
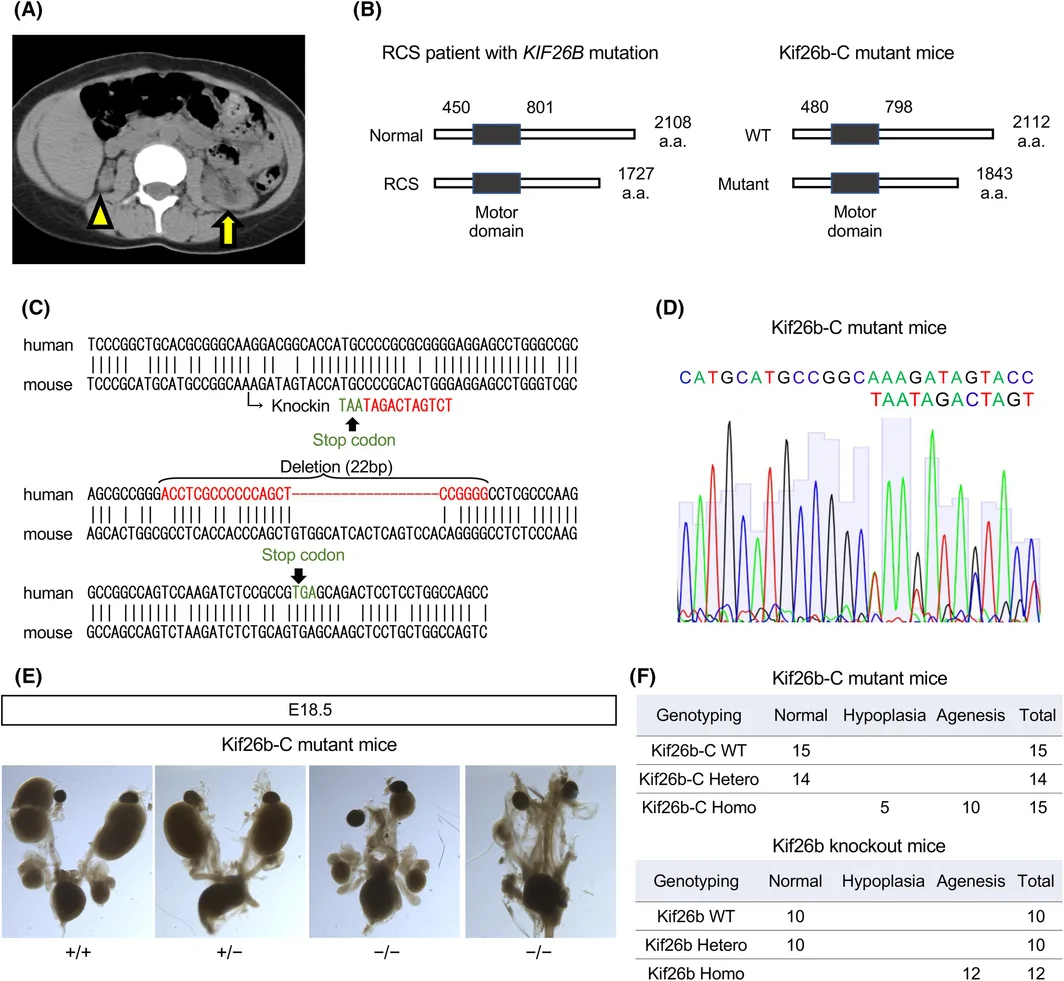
The C‐terminal region of KIF26B is essential for kidney development. (A) Representative kidney images from a renal coloboma syndrome (RCS) patient carrying a KIF26B mutation. (B) Schematic comparison of KIF26B structure between the RCS patient and the generated Kif26b mutant mice. (C) Comparison of KIF26B genomic sequences between the RCS patient and the Kif26b mutant mice. Black arrows and green text indicate the location of the stop codon. (D) Sanger sequencing of the Kif26b genomic region from Kif26b mutant mice. (E) Representative images of embryonic kidneys at embryonic day 18.5. (F) Kidney phenotype of Kif26b knockout mice.

To assess the developmental consequences of this C‐terminal truncation *in vivo*, we next examined the phenotype of Kif26b‐C mice. Homozygous Kif26b‐C mice died shortly after birth. At embryonic day 18.5 (E18.5), 10 out of 15 Kif26b‐C embryos exhibited bilateral renal agenesis, whereas the remaining five displayed unilateral agenesis with contralateral hypoplasia (Fig. 1E). These phenotypes closely resembled those of Kif26b knockout mice described previously (Fig. 1F) [3]. Furthermore, the renal defects observed in Kif26b‐C mice were similar to those seen in the human case with the KIF26B mutation (Fig. 1A). Collectively, these results indicate that the C‐terminal region of Kif26b is essential for proper kidney development in both mice and humans.

### Kif26b mutant kidneys exhibit impaired condensation of nephron progenitor cells

Next, we assessed the influence of the C‐terminal mutation in Kif26b by evaluating histology at E18.5. H&E staining of WT and heterozygous Kif26b‐C mice demonstrated a distinct nephrogenic zone, where numerous condensing mesenchymal cells and developing nephron structures, including renal vesicles, comma‐shaped, and S‐shaped bodies were observed (Fig. 2A). In contrast, homozygous Kif26b‐C mice lacked a defined nephrogenic zone, showing no condensed mesenchymal cells (Fig. 2A). We also evaluated markers of nephron progenitor cells (Integrin α8), ureteric buds (Cytokeratin 8: CK8), and both (Pax2) among WT, heterozygous, and homozygous Kif26b‐C mice. CK8 was normally detected in ureteric buds in all groups, whereas integrin α8–expressing nephron progenitor cells were markedly reduced in homozygous Kif26b‐C mice at E18.5. These findings suggest that the C‐terminal region of Kif26b is important for mesenchymal condensation around the ureteric buds and proper nephrogenic zone formation (Fig. 2B).

**Fig. 2:**
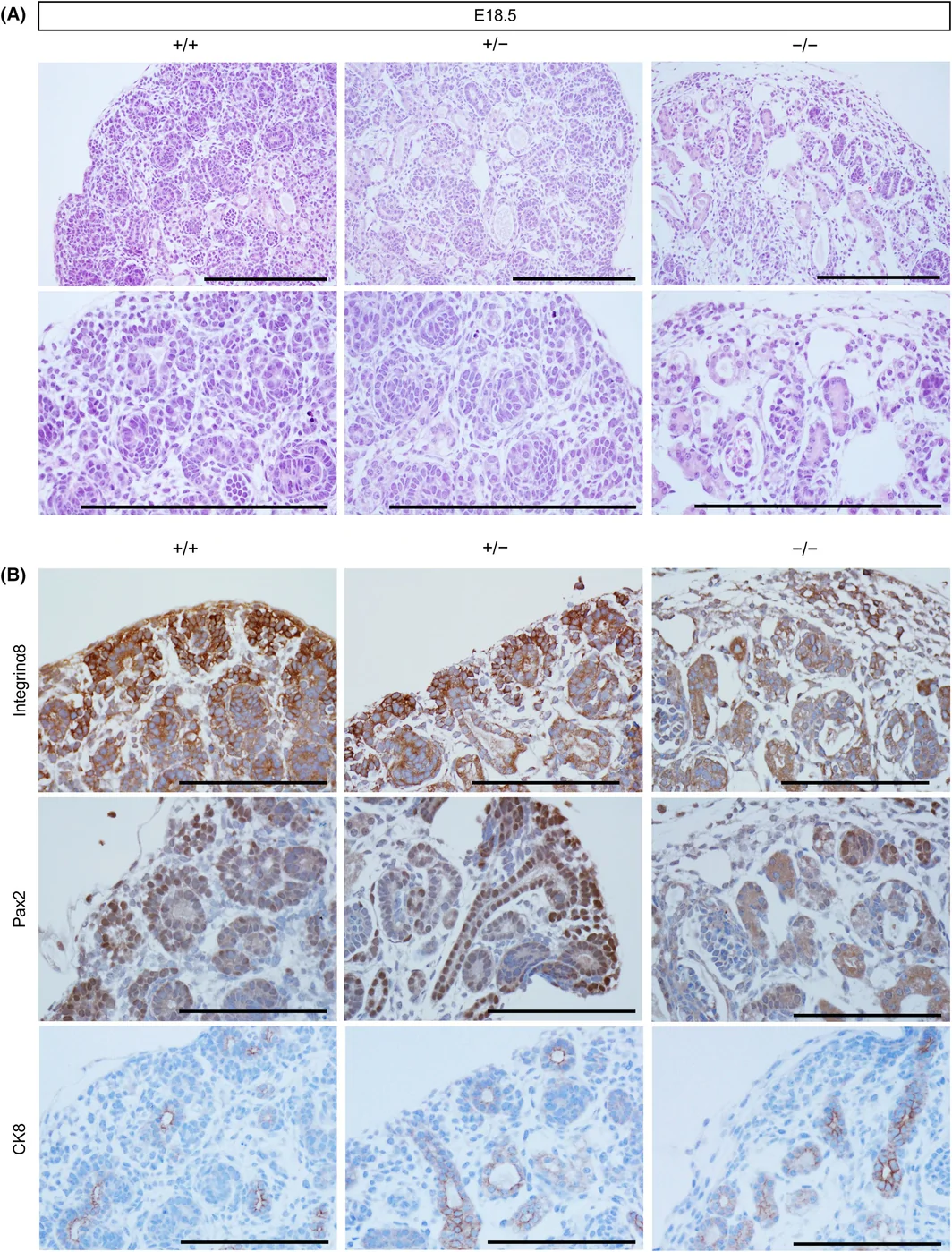
Kif26b mutant kidney impaired condensation of nephron progenitor cells. (A) Hematoxylin and eosin‐stained embryonic kidneys from WT, heterozygous Kif26b mutant, and homozygous Kif26b mutant mice at E18.5. Scale bar: 300 μm. (B) Immunostaining for Integrin α8, Pax2, and CK8 in embryonic kidneys from WT, heterozygous Kif26b mutant, and homozygous Kif26b mutant mice at E18.5. Scale bar: 300 μm.

### Deletion of the C‐terminal region of kif26b reduces Gdnf and Wnt11 expression during kidney development

To confirm the speculation about the importance of the C‐terminal region of Kif26b for condensed mesenchymal cells based on pathological findings at E18.5, we assessed Gdnf and Wnt11 expression, which are key growth factors for kidney development at an earlier developmental stage (E14.5). Gdnf, secreted from the metanephric mesenchyme, promotes ureteric bud branching, while Wnt11, secreted from the ureteric buds, enhances Gdnf expression, forming a positive feedback loop essential for kidney morphogenesis [9, 10]. *In situ* hybridization revealed that both *Gdnf* and *Wnt11* signals were strongly expressed in WT and heterozygous Kif26b‐C embryos (Fig. 3). In contrast, homozygous Kif26b‐C embryos exhibited a marked reduction in *Gdnf* expression and a more pronounced loss of *Wnt11* expression in the ureteric buds at E14.5 (Fig. 3). These results indicate altered expression of key components of the Gdnf‐Wnt11 regulatory loop in Kif26b‐C embryos.

**Fig. 3:**
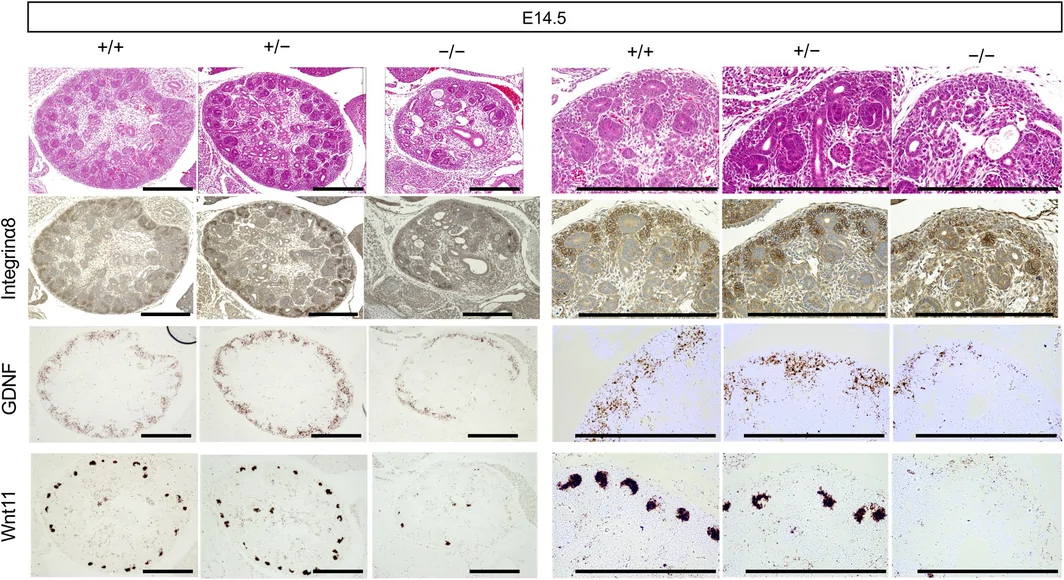
Deletion of the C terminus in kif26b exhibit impaired Gdnf‐Wnt11 signal during kidney development. H&E staining and Integrin α8 immunostaining of embryonic kidneys from WT, heterozygous Kif26b mutant, and homozygous Kif26b mutant mice at E14.5. *In situ* hybridization for Gdnf and Wnt11 in embryonic kidneys from WT, heterozygous Kif26b mutant, and homozygous Kif26b mutant mice at E14.5. Scale bar: 300 μm.

### The C‐terminal region of Kif26b is essential but not sufficient for kidney development

Lastly, to determine whether the C‐terminal region of Kif26b alone is sufficient for normal kidney development, we generated Kif26b C‐terminal region Tg mice (Kif26b Tg^C‐term^ mice) by a transgenic technique using pronuclear microinjection (Fig. 4A and Fig. S2). Expression of the C‐terminal domain in these mice was confirmed using a GFP tag, demonstrating that the transgene could produce the corresponding protein in fertilized egg injected Kif26b‐C GFP plasmid and embryonic kidneys at embryonic day 18.5 (Fig. 4B,C). This Kif26b Tg^C‐term^ mice could develop and demonstrated normal kidney morphology at E18.5 and adult mice (Fig. 4D). To generate animals expressing only the C‐terminal domain of Kif26b, we crossed the Kif26b Tg^C‐term^ mice with Kif26b knockout mice. The resulting offspring displayed kidney agenesis similar to that of Kif26b knockout mice and died perinatally (Fig. 4E). These results indicate that although the C‐terminal domain of Kif26b is expressed, its expression alone is insufficient to support kidney development.

**Fig. 4:**
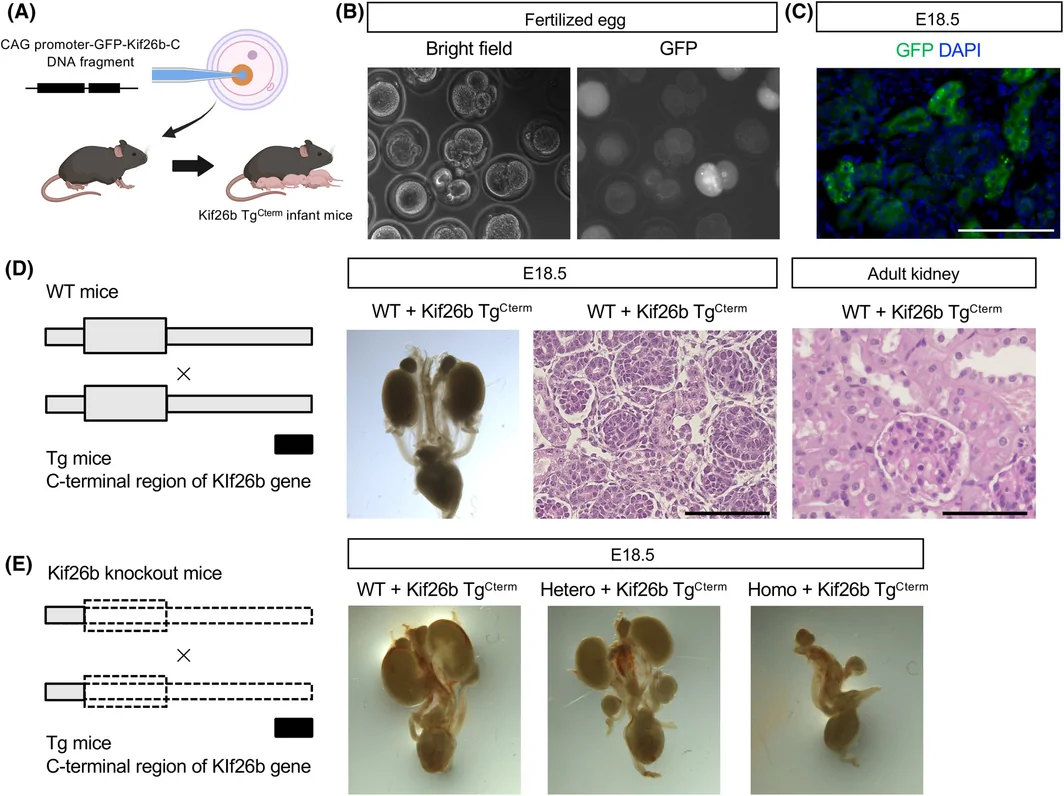
The C‐terminal region of Kif26b is essential but not sufficient for kidney development. (A) Schematic cartoon of generating Kif26b Tg^Cterm^. (B) GFP expression in fertilized egg injected Kif26b‐C GFP plasmid. (C) GFP expression of embryonic kidneys of Kif26b Tg^Cterm^ at embryonic day 18.5. Scale bar: 100 μm. (D) Schematic cartoon of evaluated mice and representative images and H&E staining of embryonic kidneys at embryonic day 18.5 and H&E staining of adult kidney. Scale bar: 100 μm. (E) Schematic cartoon of evaluated mice and representative images of embryonic kidneys at embryonic day 18.5. Created in biorender. Yamamura, Y. (2025) https://BioRender.com/tn5r2ys.

## Discussion

In this study, we sought to clarify how the C‐terminal domain of Kif26b contributes to kidney morphogenesis in both humans and mice. Our findings demonstrate that the C‐terminal region is indispensable for normal kidney development, as its deletion results in severe renal defects closely phenocopying those observed in patients with RCS. Homozygous Kif26b‐C mice exhibited bilateral or unilateral renal agenesis, impaired condensation of nephron progenitor cells, and reduced expression of Wnt11 and Gdnf. These findings indicate that the C‐terminal region of Kif26b is essential for nephrogenesis and may contribute to proper reciprocal signaling between the ureteric bud and metanephric mesenchyme.

Previous studies have established that Kif26b regulates kidney development by promoting cell adhesion within the metanephric mesenchyme [1]. Our present findings extend this understanding by pinpointing the C‐terminal domain as a critical functional determinant. Consistent with the predominant expression of Kif26b in the metanephric mesenchyme, we observed a marked defect in the formation of the integrin α8–positive metanephric mesenchyme, whereas CK8‐positive ureteric buds appeared comparatively preserved. The observed reduction in Gdnf and Wnt11 expression in Kif26b‐C embryos suggests that the C‐terminal domain contributes to proper signaling between nephron progenitors and ureteric buds, possibly through interactions with cytoskeletal components such as Myh10, as previously suggested [1, 4]. These changes may reflect impaired communication between the metanephric mesenchyme and ureteric bud tips. For example, defective mesenchymal condensation could contribute to reduced Wnt11 and Gdnf expression. However, because our analyses were performed at relatively late stages (E14.5–E18.5), we cannot distinguish whether these changes arise from primary defects in ureteric bud tip–cap mesenchyme interactions or represent secondary consequences of earlier developmental abnormalities; analyses at earlier stages will be required to address this point.

Interestingly, expression of the C‐terminal domain alone was insufficient to rescue kidney development in Kif26b‐null mice, despite being properly expressed in transgenic animals. This finding indicates that while the C‐terminal region is necessary, the presence of other regions of Kif26b, including the motor domain, is required for the full functionality of the protein. This observation aligns with the notion that kinesins often require coordinated activity of multiple domains to mediate intracellular transport and cytoskeletal organization, which are essential for tissue morphogenesis [2].

Expression of an isolated protein domain can, in principle, interfere with endogenous protein function by competing for shared binding partners. However, Kif26b Tg^C‐term^ mice did not display an overt renal phenotype despite detectable expression of the GFP‐tagged fragment, suggesting that the C‐terminal region alone is unlikely to perturb kidney development under our conditions.

Several limitations should be acknowledged. First, although our data indicate that the C‐terminal domain is essential, the precise molecular interactions mediating its function remain to be defined. Future studies employing protein interaction assays or high‐resolution transcriptome analysis via scRNA‐seq may help elucidate how the C‐terminal domain regulates mesenchymal condensation and associated signaling pathways. Second, while our mouse model recapitulates human RCS phenotypes, differences in developmental timing and compensatory mechanisms between species may exist [11, 12]. Third, in this study, sex was not considered as a biological variable. Therefore, we cannot exclude the possibility that sex‐related differences contributed to the observed phenotypic variability (bilateral renal agenesis versus unilateral agenesis with hypoplasia). Future studies focusing on sex differences will be required to address this point. Nevertheless, our findings provide a framework for understanding how KIF26B mutations contribute to congenital kidney hypoplasia in humans.

In summary, our study establishes the C‐terminal domain of Kif26b as a critical determinant of kidney development, necessary for mesenchymal condensation and associated Gdnf/Wnt11 expression patterns during nephrogenesis. These results advance our understanding of the molecular mechanisms underlying renal hypoplasia and may inform future therapeutic strategies for congenital kidney disorders caused by KIF26B mutations.

## Conflict of interest

The authors declare no conflict of interest.

## Author contributions

YY designed and performed experiments, analyzed and interpreted data, and drafted the manuscript. HA, NY, SK, AK, TM, MO, AH, NS, MS, KT, MY, and YS performed experiments. SH and TD generated genetically modified mice. HK, KH, and TW critically revised the manuscript. KF and YI critically revised and finalized the manuscript.

## Supporting information


**Fig. S1.** Generation of Kif26b mutant mice with the deletion of C‐terminal region of Kif26b.
**Fig. S2.** Generation of Kif26b C‐terminal region transgenic mice.

## Data Availability

The data that support the findings of this study are available from the corresponding author upon reasonable request.

## References

[1] Miki H , Setou M , Kaneshiro K and Hirokawa N (2001) All kinesin superfamily protein, KIF, genes in mouse and human. Proc Natl Acad Sci USA 98, 7004–7011. 10.1073/pnas.111145398 PMC34614 11416179

[2] Hirokawa N , Noda Y , Tanaka Y and Niwa S (2009) Kinesin superfamily motor proteins and intracellular transport. Nat Rev Mol Cell Biol 10, 682–696. 10.1038/nrm2774 19773780

[3] Uchiyama Y , Sakaguchi M , Terabayashi T , Inenaga T , Inoue S , Kobayashi C , Oshima N , Kiyonari H , Nakagata N , Sato Y *et al*. (2010) Kif26b, a kinesin family gene, regulates adhesion of the embryonic kidney mesenchyme. Proc Natl Acad Sci USA 107, 9240–9245. 10.1073/pnas.0913748107 PMC2889100 20439720

[4] Okumura T , Furuichi K , Higashide T , Sakurai M , Hashimoto S , Shinozaki Y , Hara A , Iwata Y , Sakai N , Sugiyama K *et al*. (2015) Association of PAX2 and other gene mutations with the clinical manifestations of renal Coloboma syndrome. PLoS One 10, e0142843. 10.1371/journal.pone.0142843 PMC4646464 26571382

[5] Recuenco MC , Ohmori T , Tanigawa S , Taguchi A , Fujimura S , Conti MA , Wei Q , Kiyonari H , Abe T , Adelstein RS *et al*. (2015) Nonmuscle myosin II regulates the morphogenesis of Metanephric mesenchyme‐derived immature nephrons. J Am Soc Nephrol 26, 1081–1091. 10.1681/ASN.2014030281 PMC4413762 25168025

[6] Terabayashi T , Sakaguchi M , Shinmyozu K , Ohshima T , Johjima A , Ogura T , Miki H and Nishinakamura R (2012) Phosphorylation of Kif26b promotes its polyubiquitination and subsequent proteasomal degradation during kidney development. PLoS One 7, e39714. 10.1371/journal.pone.0039714 PMC3387196 22768111

[7] Yamamura Y , Iwata Y , Furuichi K , Kato T , Yamamoto N , Horikoshi K , Ogura H , Sato K , Oshima M , Nakagawa S *et al*. (2022) Kif26b contributes to the progression of interstitial fibrosis via migration and myofibroblast differentiation in renal fibroblast. FASEB J 36, e22606. 10.1096/fj.202200355R 36250931

[8] Karuna E , Choi S , Scales M , Hum J , Cohen M , Fierro F and Ho H‐Y (2018) Identification of a WNT5A‐responsive degradation domain in the kinesin superfamily protein KIF26B. Gen 9, 196. 10.3390/genes9040196 PMC5924538 29621187

[9] Little MH and McMahon AP (2012) Mammalian kidney development: principles, progress, and projections. Cold Spring Harb Perspect Biol 4, a008300. 10.1101/cshperspect.a008300 PMC3331696 22550230

[10] Schnell J , Achieng M and Lindström NO (2022) Principles of human and mouse nephron development. Nat Rev Nephrol 18, 628–642. 10.1038/s41581-022-00598-5 PMC12980996 35869368

[11] Lindström NO , Guo J , Kim AD , Tran T , Guo Q , De Sena Brandine G , Ransick A , Parvez RK , Thornton ME , Baskin L *et al*. (2018) Conserved and divergent features of mesenchymal progenitor cell types within the cortical nephrogenic niche of the human and mouse kidney. J Am Soc Nephrol 29, 806–824. 10.1681/ASN.2017080890 PMC5827607 29449449

[12] Lindström NO , Tran T , Guo J , Rutledge E , Parvez RK , Thornton ME , Grubbs B , McMahon JA and McMahon AP (2018) Conserved and divergent molecular and anatomic features of human and mouse nephron patterning. J Am Soc Nephrol 29, 825–840. 10.1681/ASN.2017091036 PMC5827611 29449451

