# Supplementary material for: The C‐terminal region of KIF26B is indispensable for nephron progenitor condensation and kidney formation in mice
Source: FEBS Open Bio. 2026 May 18:10.1002/2211-5463.70260. Online ahead of print. doi: 10.1002/2211-5463.70260 (PMC13398707; doi:10.1002/2211-5463.70260)
Supplement: Supplementary file 1 — Fig. S1. Generation of Kif26b mutant mice with the deletion of C‐terminal region of Kif26b. Fig. S2. Generation of Kif26b C‐terminal region transgenic mice. [file FEB4-9999-0-s001.pdf]

Supplemental Figure 1

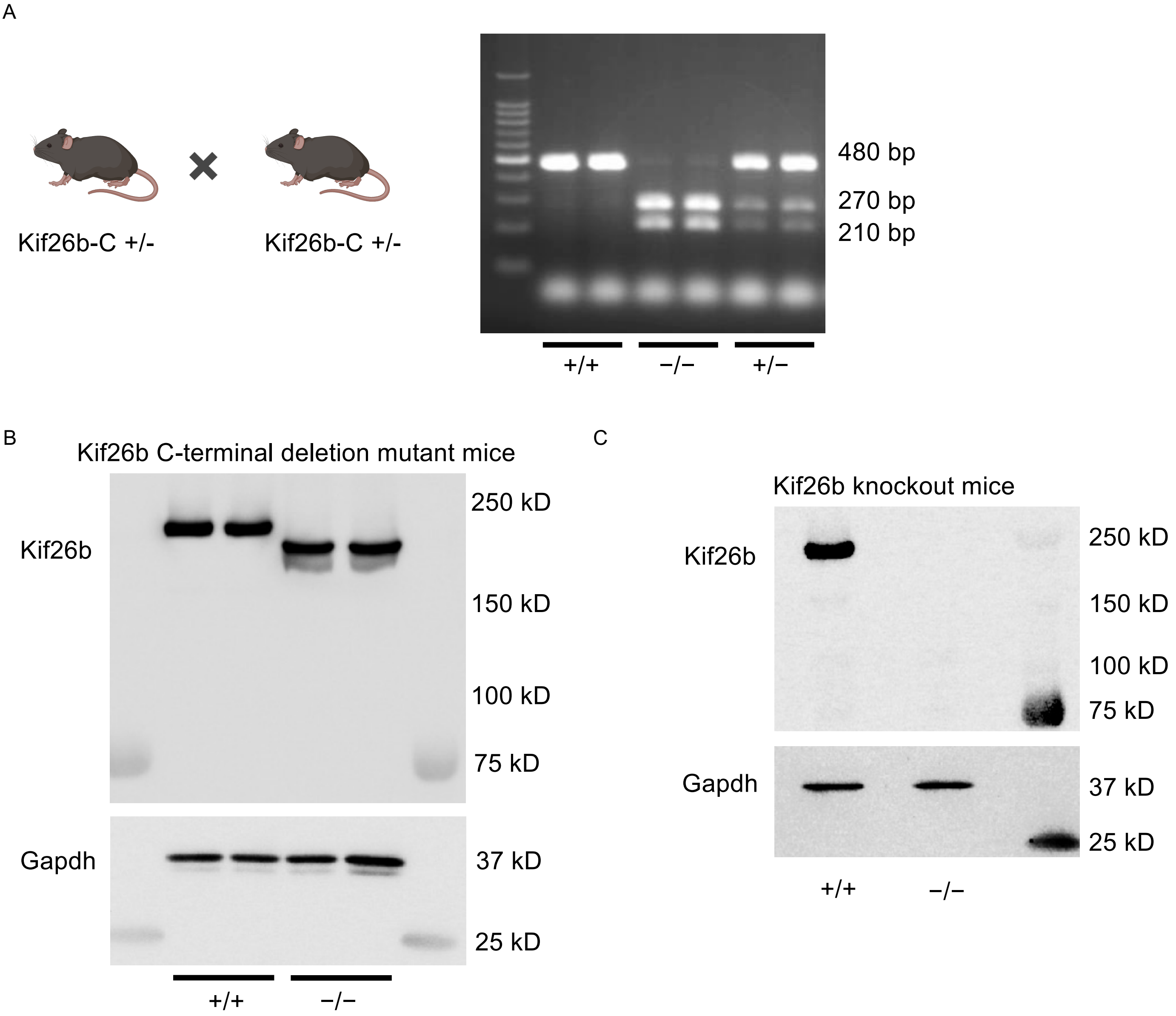

Supplemental Figure 1. Genotyping strategy for Kif26b mutant mice with the deletion of C-terminal region of Kif26b. (A) Schematic images and representative PCR genotyping results. (B) KIF26B protein expression in embryonic kidneys from WT and Kif26b C-terminal deletion mutant mice at E14.5. (C) KIF26B protein expression in limb buds from WT and Kif26b knockout mice at E12.5. Created in BioRender. Yamamura, Y. (2025) https://BioRender.com/tn5r2ys.

Supplemental Figure 2

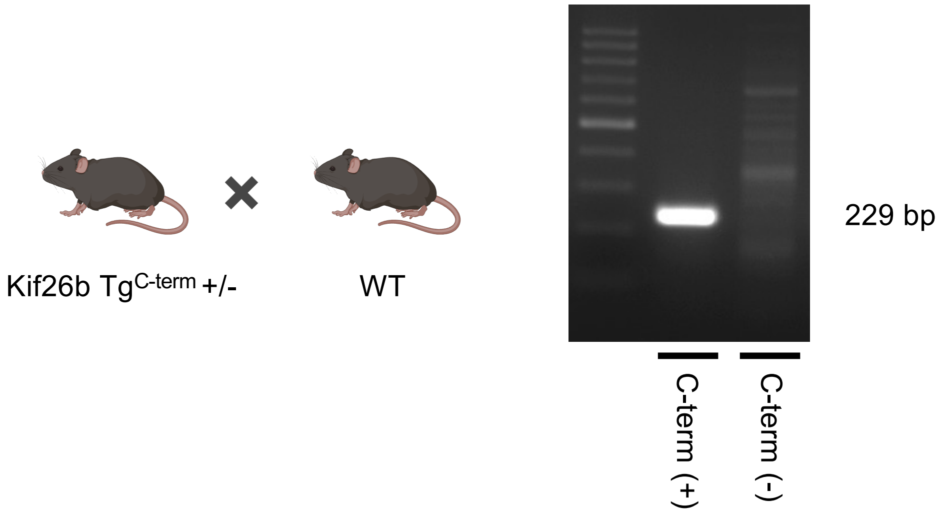

Supplemental Figure 2. Genotyping strategy for Kif26b C-terminal region transgenic mice.
Created in BioRender. Yamamura, Y. (2025) https://BioRender.com/tn5r2ys.
